# Chagas vectors *Panstrongylus chinai* (Del Ponte, 1929) and *Panstrongylus howardi* (Neiva, 1911): chromatic forms or true species?

**DOI:** 10.1186/s13071-020-04097-z

**Published:** 2020-05-06

**Authors:** Anita G. Villacís, Jean-Pierre Dujardin, Francisco Panzera, César A. Yumiseva, Sebastián Pita, Soledad Santillán-Guayasamín, Marco I. Orozco, Katherine D. Mosquera, Mario J. Grijalva

**Affiliations:** 1 Center for Research on Health in Latin America (CISeAL), School of Biological Sciences, Pontificia Universidad Católica del Ecuador, Av. 12 de Octubre 1076 y Roca, Quito, Ecuador; 2 Infectious and Tropical Disease Institute, Department of Biomedical Sciences, Heritage College of Osteopathic Medicine, Ohio University, Athens, OH 45701 USA; 3 IRD, UMR 177 IRD-CIRAD INTERTRYP, Campus international de Baillarguet, Montpellier, France; 4 Sección Genética Evolutiva, Facultad de Ciencias, Universidad de la República, Montevideo, Uruguay; 5 Carrera de Ingeniería en Biotecnología, Departamento de Ciencias de la Vida y la Agricultura, Universidad de las Fuerzas Armadas - ESPE, Sangolquí, Ecuador

**Keywords:** Antennal phenotype, Experimental hybridization, Ecological niche modeling, Geometric morphometry, *Panstrongylus*, Triatominae, Ecuador

## Abstract

**Background:**

Chagas disease is a parasitic infection transmitted by “kissing bugs” (Hemiptera: Reduviidae: Triatominae) that has a huge economic impact in Latin American countries. The vector species with the upmost epidemiological importance in Ecuador are *Rhodnius ecuadoriensis* (Lent & Leon, 1958) and *Triatoma dimidiata* (Latreille, 1811). However, other species such as *Panstrongylus howardi* (Neiva, 1911) and *Panstrongylus chinai* (Del Ponte, 1929) act as secondary vectors due to their growing adaptation to domestic structures and their ability to transmit the parasite to humans. The latter two taxa are distributed in two different regions, they are allopatric and differ mainly by their general color. Their relative morphological similarity led some authors to suspect that *P. chinai* is a melanic form of *P. howardi*.

**Methods:**

The present study explored this question using different approaches: antennal phenotype; geometric morphometrics of heads, wings and eggs; cytogenetics; molecular genetics; experimental crosses; and ecological niche modeling.

**Results:**

The antennal morphology, geometric morphometrics of head and wing shape and cytogenetic analysis were unable to show distinct differences between the two taxa. However, geometric morphometrics of the eggs, molecular genetics, ecological niche modeling and experimental crosses including chromosomal analyses of the F1 hybrids, in addition to their coloration and current distribution support the hypothesis that *P. chinai* and *P. howardi* are separate species.

**Conclusions:**

Based on the evidence provided here, *P. howardi* and *P. chinai* should not be synonymized. They represent two valid, closely related species.
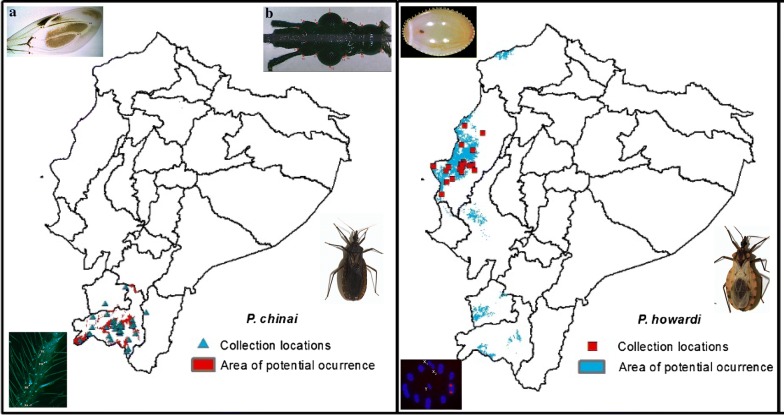

## Background

Chagas disease constitutes a serious problem in Latin America. Today, six to seven million people worldwide, mostly in Latin America, are infected with *Trypanosoma cruzi*, the causative agent of this disease [1]. The population most commonly at risk is typically of low socioeconomic status [2]. The main way of transmission is through the contact with infected feces of insects of the subfamily Triatominae, so vector control is the most useful method to prevent Chagas disease in Latin America [3].

Currently, 16 triatomine species have been reported in Ecuador, with *Rhodnius ecuadoriensis* and *Triatoma dimidiata* being the main Chagas disease vectors [4, 5]. Six species of the genus *Panstrongylus* have also been reported in Ecuador: *Panstrongylus chinai*; *P. howardi*; *P. geniculatus* (Latreille,1811); *P. lignarius*/*P. herreri* (Walker, 1873); and *P. rufotuberculatus* (Champion, 1899) [6, 7]. The epidemiological importance of several of these species, considered to act as secondary vectors, has been dramatically increased by their invasion into human structures [8]. Some species of *Panstrongylus* have a high capacity as Chagas disease vectors because of their longevity, rapid response to the presence of a host, the large volume of blood ingested, and frequent defecation during the feeding process [9].

*Panstrongylus chinai* is distributed in Loja and El Oro provinces, in the southern Andean region of Ecuador. This species has also been reported in the north of Peru, on the border with Loja, in different wild habitats and colonizing human dwellings [6]. In Ecuador, the species is found in domestic and peridomestic environments such as chicken coops, pigeon and rat nests. Inside houses, it is found mainly under beds or in holes in the walls. In Loja (Ecuador) and some areas of Peru, *P. chinai* has been categorized as a potential vector of Chagas disease requiring continuous entomological surveillance [9].

*Panstrongylus howardi* is an endemic species for Ecuador, restricted to the Manabí Province (central coast region) [6, 7]. This species has been found only once in wild conditions [10] but is frequently collected in peridomestic habitats associated with rodent nests located between brick piles [6, 7, 11]. Abundant colonies can be also found in wood piles, as well as in the “piñuelas” plant *Aechmea magdalenae*, again in association with nesting places of rodents or marsupials [12]. *Panstrongylus howardi* shows high levels of infection with *Trypanosoma* parasites (*T. cruzi* and *T. rangeli*), which suggests the existence of active transmission between this vector species and its associated vertebrate hosts [8].

*Panstrongylus chinai* and *P. howardi* have been regarded as belonging to the same evolutionary clade [3, 13]. Their morphological similarity has led some authors to suspect *P. chinai* to be a melanic form of *P. howardi* [13, 14]. The present study aims to explore this question through an integrative approach including quantitative morphology, chromosome analyses, molecular genetics, mating behavior and ecological arguments.

Integrative taxonomy has received considerable attention in the last years as a multidisciplinary approach that aims at delimiting the units of life’s diversity from multiple and complementary perspectives [15, 16]. It is useful to evaluate the species limits and is of great help in cases of recent speciation processes, cryptic diversity, or simply intraspecific variation (phenotypic plasticity) [17–19]. Historically, taxonomists have been concerned with classifying organisms into groups based on shared traits, and then further classifying those groups into the categories of the taxonomic hierarchy, from kingdom to species. In contrast, modern systematic biologists, despite the fact that they still use data taking the same basic form of similarities and differences among organisms, are increasingly devoting their efforts to testing hypotheses about lineage boundaries and phylogenetic relationships [20]. A review of methods used in taxonomic studies related to the Triatominae concluded that an integrative approach was necessary, putting forward the concept of “evolutionary lineage” [21]. The evolutionary concept allows the discussion about infraspecific populations and decisions about the utility of giving them subspecific ranks [22]. It was applied successfully to various questions related to speciation in the subfamily Triatominae [23–26]. Current taxonomy of the Triatominae has been examined in the light of the main species concepts (morphological, biological, Hennigian, phylogenetic and evolutionary concepts) by Bargues et al. [19, 21].

Morphological variation in the Triatominae is clearly modulated by ecological factors. Features like color patterns or pilosity can change within a single species when populations are under disruptive selection or collected in a wide geographical range, a common observation which has been attributed to apparently weak canalization mechanisms [26]. Developmental canalization mechanisms maintain the stability of a given phenotype in the face of environmental disturbance. The existence of distinct forms, morphs or ecotypes in many species is indicative of a weak developmental canalization in the Triatominae [26]. This has been quantified between populations or among different species by studies on the size, shape (head or wing), or on the number of sensorial receptors (sensilla) covering the antennae [27–32]. In the Triatominae, the morphological variation of eggs among species and populations has also been used in qualitative [33–37] and quantitative analyses [38–42]. In this study, we examined the geometric morphometrical variation of both adults and eggs of these two species.

Chromosomal studies have been applied extensively in this subfamily to differentiate species or determine common characteristics in evolutionarily close species. Chromosomal variations in the amount of constitutive heterochromatin and in the position of the ribosomal clusters are the main intraspecific and interspecific features for karyotypic differentiation in the Triatominae [43, 44].

Molecular tools applied to Ecuadorian triatomines have been poorly explored [45]. The mitochondrial *cytochrome b* (*cytb*) gene has been found to be a very useful molecular marker to differentiate cryptic species or closely related species in different triatomine genera [46–48].

Experimental crosses (EC) had an important role in clarifying controversial taxonomic issues within the Triatominae [49–55]. Hybridization studies may be very helpful to formulate hypotheses concerning the origin and divergence of species [56]. In case of allopatry, it is not possible to ascertain that the hybridization (or lack of hybridization) observed in the laboratory would be indeed the natural behavior of these taxa if they could meet in natural conditions. Reproductive isolation, even when observed experimentally, is one of the best criteria to evaluate the taxonomic status of morphologically or genetically close populations [56].

A complementary approach to the systematics of the Triatominae has been the recent application of ecological niche modeling (ENM) [57]. The ENM is a method that predicts the potential geographical distribution of a species under certain environmental conditions, even in territories where the species may have not reached yet [57–61]. The known distribution of *P. howardi* and *P. chinai* is restricted to separate territories in Ecuador, so that it is relevant to explore their potential geographical distribution. *Panstrongylus chinai* occurs in an arid zone (Loja), located in a wide range of altitudes (175 to 2003 meters above sea level, masl) [62], while *P. howardi* is restricted to the more humid and much lower (up to 400 masl) territory of the Manabí Province [10, 63].

The present study tries to discern if *P. howardi* and *P. chinai* are valid species or are two populations of the same species with morphological variations, mainly color and size differences, as proposed by Abad-Franch & Aguilar [14] and Patterson et al. [13]. In the Triatominae, it has been shown that morphological divergence may occur rapidly, before the establishment of reproductive barriers [64]. If this is the case, *P. chinai* and *P. howardi* should not, or barely, depart from what is expected between populations of the same species when compared through diverse biological analyses. For this reason, to demonstrate if *P. chinai* and *P. howardi* are chromatic forms or true species, the following approaches were used: comparative assessment of the antennal phenotype (APH); geometric morphometrics (GM); cytogenetics (CYT); molecular genetics (MG); experimental crosses (EC) including chromosomal analyses of the hybrids; and ecological niche modeling (ENM).

## Methods

### Study area and triatomine collection

**Fig. 1:**
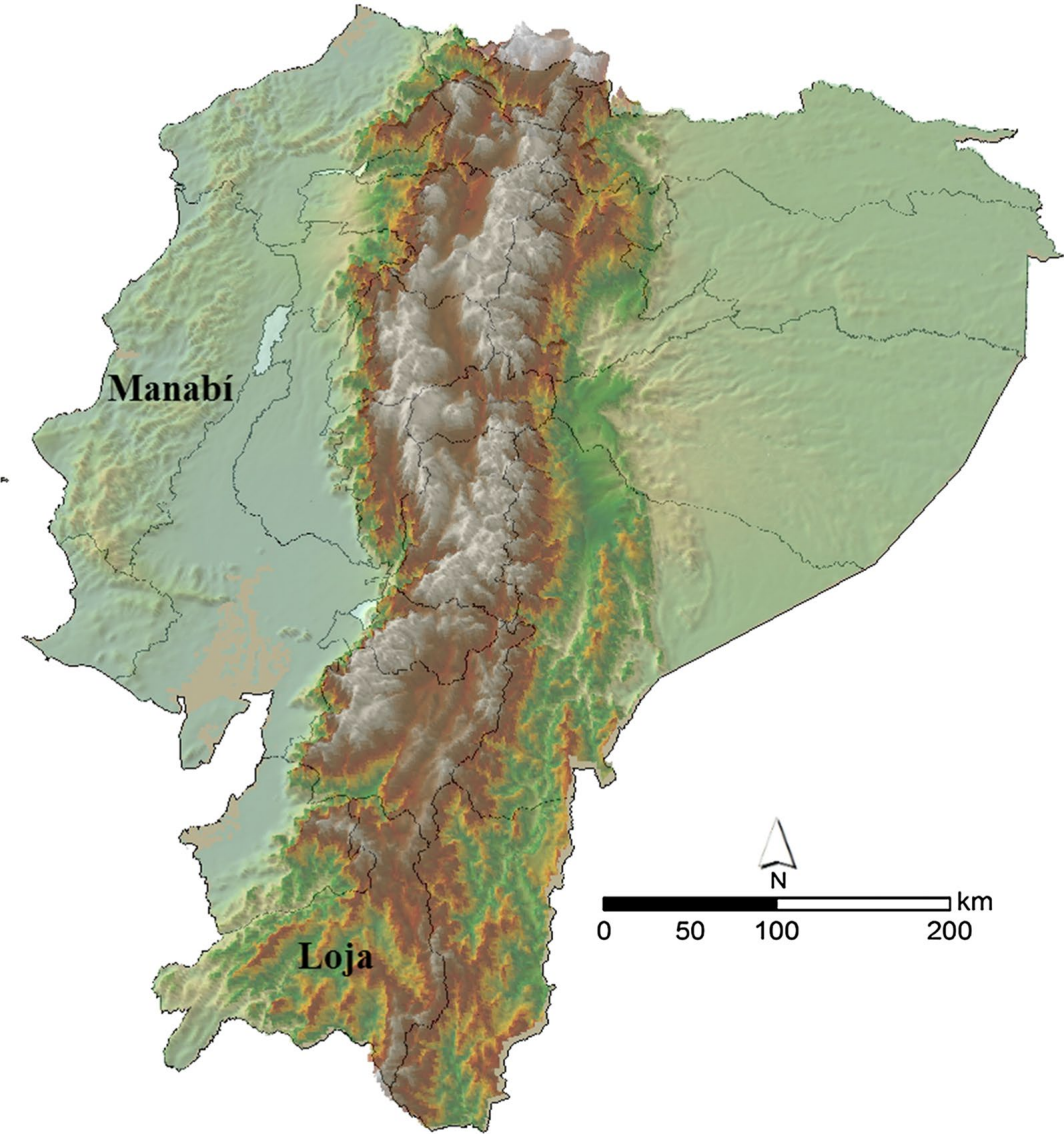
Map of Ecuador. The Andes Mountains cross the Ecuador from North to South, dividing the continental territory in three natural regions: Coastal Region, where the Manabí Province belongs; southern Andean Region, where the Loja Province belongs; and the Amazonia or Eastern Region

In Ecuador, specimens under study are exclusively found in the Central Coastal region (*P. howardi*, Manabí Province) and in the southern Andean Region (*P. chinai*, Loja Province) (Fig. 1). The triatomines were collected in 11 communities of Manabí and 17 localities of Loja during the years 2004–2011 (Additional file 1: Table S1). Both species were collected in domestic and peridomestic habitats in Loja and Manabí provinces as described by Grijalva et al. [65] and Villacís et al. [7], respectively. The domestic searches included all the rooms inside each house. The peridomestic habitat was defined as all surrounding structures and potential microhabitats within 20 m of the house (including chicken coops and pigeon nests; accumulations of stones, tiles, wood, bricks, piles of agricultural products, storage buildings; and other structures near the houses).

Manabí Province is located along the Central Coast of Ecuador at an altitude ranging from 0 to 400 masl, and receives an average annual rainfall of 563 mm [66]. The walls and floors of houses in this region are principally constructed with bamboo cane “caña guadúa” (*Guadua angustifolia*) or wood, the roofs are commonly made of the cade palm frowns, and in less proportion made of zinc [67].

The Province of Loja, in the southern Andean region of Ecuador, is characterized by a mix of hilly and mountainous topography, with an altitude ranging from 120 to 3800 masl. The region includes inter-Andean temperate valleys and has an average rainfall of 400 mm [66]. Houses are typically made of adobe walls, the floors with dirt, and the roofs are made with ceramic tile [65, 67, 68].

### Morphology

#### Analysis of antennal phenotype

**Fig. 2:**
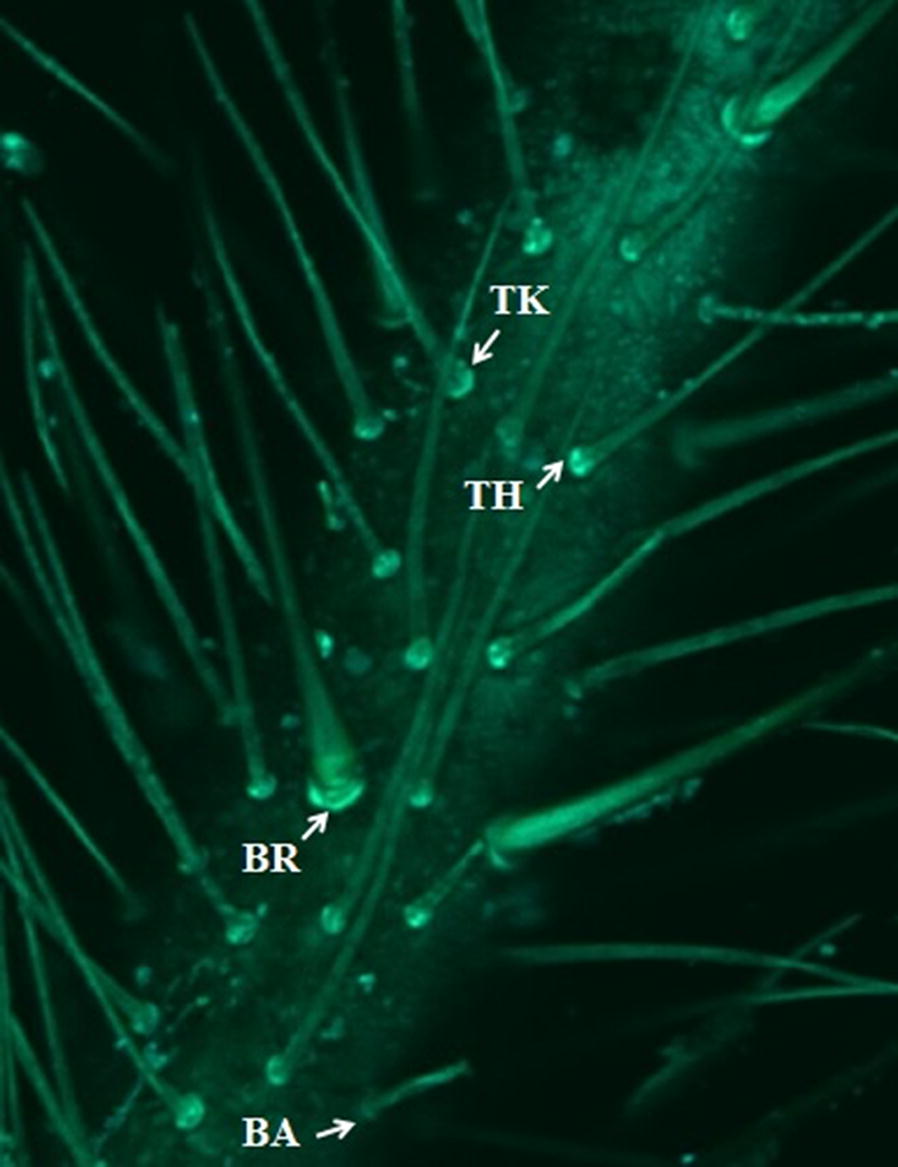
Antennal phenotype (APH) of *Panstrongylus chinai* (SEM micrograph). The APH represents the number and type of sensilla in each segment of the antenna. Sensilla are: bristles (BR); thin-walled trichoidea (TH); thick-walled trichoidea (TK); and basiconic (BA)

The antennae of 44 adult *P. chinai* (15 females and 29 males) and 23 *P. howardi* (8 females and 15 males) were analyzed following the methodology of Abrahan et al. [69]. One antenna was cut from each individual at the level of the scape (segment 1), and slide-mounted in glycerine. By optical microscopy at 400× (Olympus BX41) with a lucid camera (Olympus U-DA 9E12246; Olympus corporation, Tokyo, Japan), the number of sensilla of different types was counted over the whole ventral surface of the three distal segments of the antenna, i.e. the pedicel (Pedi), and the two flagellar segments: flagellum 1 (Flag1) and flagellum 2 (Flag2). For this study, we considered the mechanoreceptors (bristles, BR) and three types of chemoreceptors: thin-walled trichoidea (TH); thick-walled trichoidea (TK); and basiconics (BA) (nomenclature following Catalá & Schofield [70] (Fig. 2). For each segment, the number of each kind of sensilla per ventral face (antennal phenotype) was used for statistical comparisons between sexes and species.

#### Geometric morphometrics

Morphometric analyses of adults and eggs followed two main steps: (i) extraction of size and shape variables; and (ii) discrimination between species or groups based on shape variables.

**Fig. 3:**
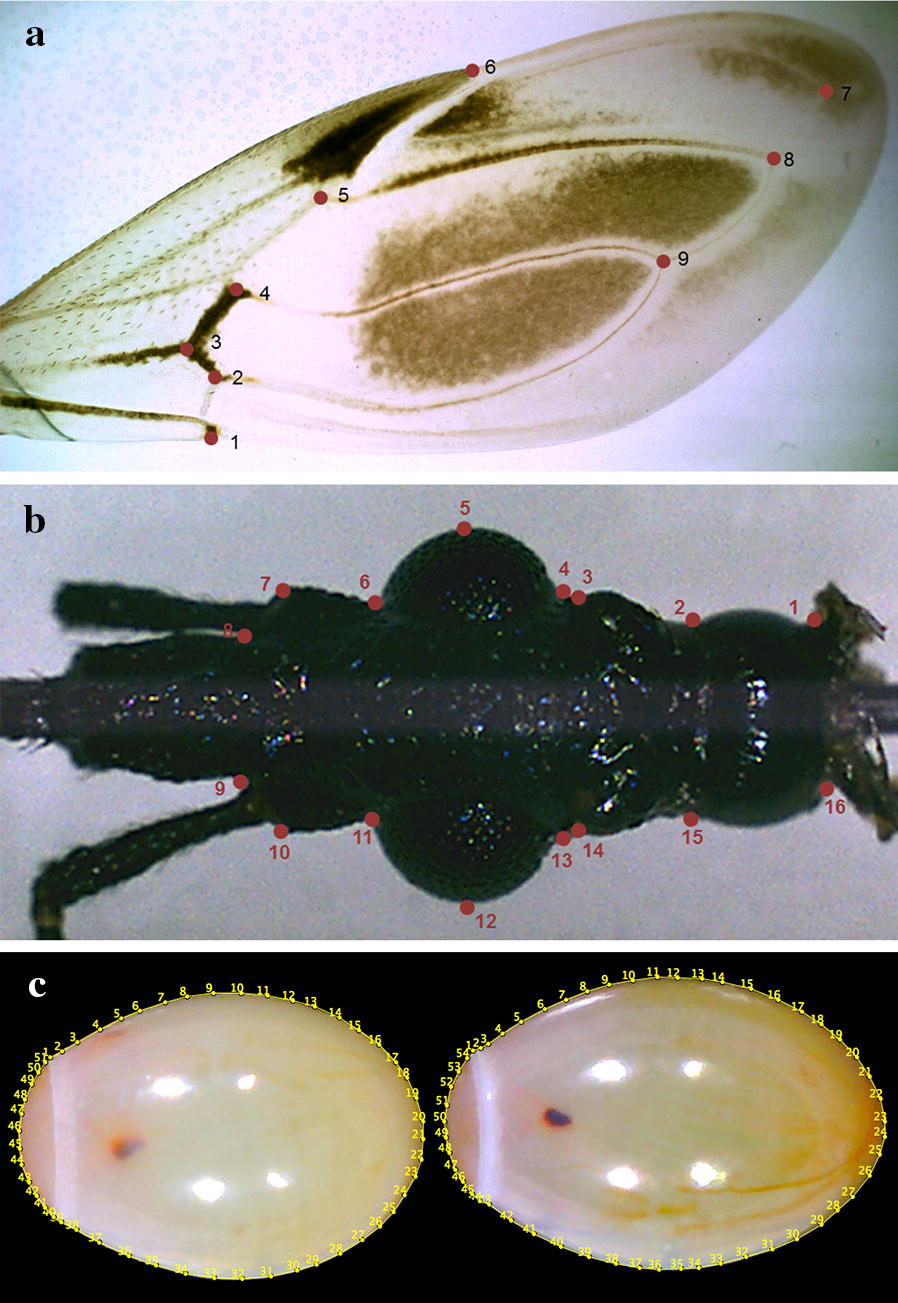
Landmarks and pseudolandmarks as digitized for wings, heads and eggs. **a** Dorsal view of the right wing of a female *P. howardi*. **b** Dorsal view of the head of a female *P. howardi*. Dots indicate the landmarks used for the analysis (wing and head). Numbers indicate the order of landmark collection. For the head, homologous landmarks of right and left sides (1 and 16, 2 and 15, 3 and 14, etc.) were averaged (after reflection of one side) for statistical analyses. **c** Eggs of *P. howardi* (left) and *P. chinai* (right), numbered points are pseudo-landmarks

*Wings and heads*: a total of 79 right hemelytra and heads of 18 females and 25 males of *P. chinai*, and of 15 females and 21 males of *P. howardi*, were prepared for geometric morphometric analyses. The wings and heads were deposited on microscope slides. The wings were cautiously placed on a coverslip, and the heads were positioned parallel to the focal plane, using an entomological pin. A digital image of each organ was obtained using a MiScope®-MIP (http://www.zarbeco.com). Nine landmarks were identified for each wing (Fig. 3a) and 16 landmarks were identified for each head (Fig. 3b). For the head, the coordinates of corresponding landmarks on the left and right sides were averaged, thus reducing the number of landmarks to 8, prior to statistical analyses. Collection of landmarks (on heads and wings) and subsequent statistical analysis were performed using the various modules of the CLIC package (version 99), written by one of us (JPD) and freely available at http://xyom-clic.eu.

**Fig. 4:**
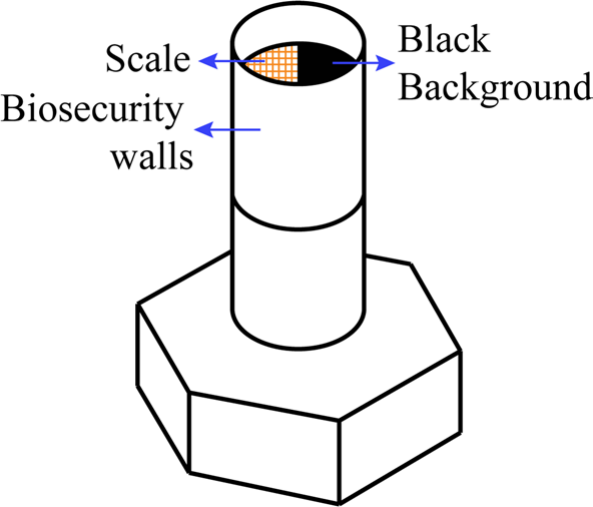
Platform device for the eggs. The platform was a bolt with, on top, a black paper (background) and a semicircular graph paper (scale), protected by a wall for biosecurity

*Eggs*: the 78 *P. chinai* eggs came from 48 females (6 localities) and the 75 *P. howardi* eggs came from 8 females (one locality) (Additional file 1: Table S1). All of them were viable eggs, obtained from virgin, laboratory females coming from field-collected specimens. To ensure a reproducible protocol of image capture (MiScope-MIP), the 153 viable eggs were photographed one by one at the same developmental time (25 days of development) and exactly the same position (ventral) on a small platform (Fig. 4), as described in Santillán-Guayasamín et al. [41]. For morphometric comparisons, we only considered the complete contour of the egg, thus including the operculum (Fig. 3c), using the outline-based approach [71, 72]. As for the landmark-based analyses of head and wings, pseudolandmarks were collected and analyzed using the CLIC package.

### Cytogenetics

Testes were removed from freshly killed adults, fixed in an ethanol-acetic acid mixture (3:1) and stored at − 20 °C. Chromosome studies were made on 2 male specimens of *P. chinai* from Loja province and 5 specimens of *P. howardi* from Manabí Province (Additional file 1: Table S1). Meiotic processes of two male F1 hybrids resulting from the cross between a male of *P. chinai* (Loja) with a female of *P. howardi* (Manabí) and the reverse cross were also analyzed. Chromosomal techniques were applied using squashed gonad preparations made on slides in a drop of 50% acetic acid, freezing them in liquid nitrogen and removing the coverslip with a razor blade. The C-banding technique was performed as reported by Panzera et al. [38] in order to observe the distribution and behavior of C-heterochromatin during mitosis and meiosis. Fluorescence *in situ* hybridization (FISH) assay was carried out according to Panzera et al. [43] in order to determine the chromosomal location of the *45S* ribosomal DNA clusters. Slides were analyzed using a Nikon Eclipse 80i epifluorescence microscope (Nikon, Kanagawa, Japan). Images were obtained with a Nikon DS-5Mc-U2 digital, cooled camera (Nikon). For analyses of cytogenetic images, we used the software IPP plus, Nikon Nis Elements 3.1 Advanced Research, and Adobe Photoshop.

### Molecular genetics

For *cytochrome b* (*cytb*) marker, newly generated sequences for 26 *P. chinai* and 33 *P. howardi* deposited in the GenBank database were employed for MG analyses (Table 1, Additional file 2: Table S2). Additionally, sequences for three *Panstrongylus* species, i.e. *P. megistus* (GenBank: KC249230, KC249231) *P. tupynambai* (GenBank: KC249233, KC249234) and *P. lutzi* (GenBank: KC249262) and three *Triatoma* species, i.e. *T. tibiamaculata* (GenBank: KC249296, KC249297), *T. brasiliensis* (GenBank: AY336524) and *T. infestans* (GenBank: AY062165) were used for comparative purposes. The two species studied here represent 20 haplotypes: 9 of *P. chinai* and 11 of *P. howardi*.

**Table 1:** GenBank accession numbers and metadata for the newly generated sequences for *Panstrongylus chinai* and *P. howardi* collected in Ecuador

GenBank ID	Species	Code	Province	Community	Haplotype number
JX400933	*P. chinai*	AH2408	Loja	Ashimingo	2
JX400934	*P. chinai*	AH2421	Loja	Ashimingo	4
JX400935	*P. chinai*	AH2445	Loja	Ashimingo	2
JX400936	*P. howardi*	BJ2737	Manabí	Bejuco	18
JX400937	*P. howardi*	BJ2745	Manabí	Bejuco	18
JX400938	*P. howardi*	BJ2771	Manabí	Bejuco	13
JX400939	*P. howardi*	BJ2997	Manabí	Bejuco	17
JX400940	*P. howardi*	BJ2998	Manabí	Bejuco	20
JX400941	*P. howardi*	BJ5612	Manabí	Bejuco	18
JX400942	*P. chinai*	BR2253	Loja	Bramaderos	2
JX400943	*P. chinai*	BR2254	Loja	Bramaderos	2
JX400944	*P. chinai*	BR2260	Loja	Bramaderos	2
JX400945	*P. chinai*	CE4586	Loja	Coamine	8
JX400946	*P. howardi*	CN1349	Manabí	La Ciénega	18
JX400947	*P. howardi*	CN1350	Manabí	La Ciénega	18
JX400948	*P. howardi*	CN1351	Manabí	La Ciénega	18
JX400949	*P. howardi*	CN1352	Manabí	La Ciénega	18
JX400950	*P. howardi*	CN1360	Manabí	La Ciénega	18
JX400951	*P. howardi*	CN1725	Manabí	La Ciénega	18
JX400952	*P. howardi*	CN3519	Manabí	La Ciénega	18
JX400953	*P. howardi*	CN3558	Manabí	La Ciénega	18
JX400954	*P. chinai*	CQ4349	Loja	Chaquizhca	7
JX400955	*P. chinai*	CQ4350	Loja	Chaquizhca	7
JX400956	*P. chinai*	CQ4426	Loja	Chaquizhca	9
JX400957	*P. chinai*	CY3007	Loja	Chirimoyos	9
JX400958	*P. chinai*	CY3008	Loja	Chirimoyos	9
JX400959	*P. chinai*	CY3087	Loja	Chirimoyos	9
JX400960	*P. chinai*	EX2668	Loja	Extensa	6
JX400961	*P. chinai*	EX2692	Loja	Extensa	9
JX400962	*P. chinai*	EX4585	Loja	Extensa	5
JX400964	*P. chinai*	GA4354	Loja	Guara	9
JX400965	*P. chinai*	GA4355	Loja	Guara	9
JX400966	*P. chinai*	HW4820	Loja	Jawai	3
JX400967	*P. chinai*	HW4821	Loja	Jawai	3
JX400968	*P. howardi*	LE2951	Manabí	La Encantada	18
JX400969	*P. howardi*	LE3346	Manabí	La Encantada	18
JX400970	*P. howardi*	LE3349	Manabí	La Encantada	18
JX400971	*P. chinai*	LU4874	Loja	Lucarqui	4
JX400972	*P. howardi*	MB2958	Manabí	Maconta abajo	18
JX400973	*P. howardi*	MB2961	Manabí	Maconta abajo	16
JX400974	*P. chinai*	ND1730	Loja	Naranjo Dulce	2
JX400975	*P. chinai*	ND2701	Loja	Naranjo Dulce	2
JX400979	*P. chinai*	SF2686	Loja	San Francisco	1
JX400980	*P. howardi*	SG1346	Manabí	San Gabriel	15
JX400981	*P. howardi*	SG1348	Manabí	San Gabriel	15
JX400982	*P. chinai*	ST3013	Loja	Santa Ester	9
JX400983	*P. chinai*	ST4584	Loja	Santa Ester	9
JX400984	*P. howardi*	TBJ1528	Manabí	Bejuco	11
JX400985	*P. howardi*	TBJ1558	Manabí	Bejuco	18
JX400986	*P. howardi*	TBJ1608	Manabí	Bejuco	18
JX400987	*P. howardi*	TBJ1621	Manabí	Bejuco	18
JX400988	*P. howardi*	TBJ1691	Manabí	Bejuco	18
JX400994	*P. howardi*	TKE2387	Manabí	Pacoche	19
JX400996	*P. howardi*	TSW2317	Manabí	Santa Rosa de las Palmas	18
JX400997	*P. howardi*	ZL2778	Manabí	Zapallo	14
JX400998	*P. howardi*	ZL2781	Manabí	Zapallo	12
JX400999	*P. howardi*	ZL2782	Manabí	Zapallo	10
JX401000	*P. howardi*	ZL2788	Manabí	Zapallo	12
JX401001	*P. howardi*	ZL2790	Manabí	Zapallo	18

### Experimental crosses

Collected insects from six communities in Loja and one community in Manabí (Additional file 1: Table S1) were maintained under suitable conditions at the CISeAL (Quito, Ecuador). This facility is equipped with a “dual chamber incubator” where the original microhabitat temperature and humidity conditions were the same as those described in Villacís et al. [73] and Santillán-Guayasamín et al. [74]. Fifth-instar nymphs (NV) of *P. chinai* and *P. howardi* were separated because virgin females were needed for crossing. Once the NV emerged to adults, both females and males were placed in plastic vials (9 × 5 × 4 cm) fitted with fan-folded Whatman filter paper to facilitate movement of specimens and absorb excess humidity. Blood meals were offered fortnightly using immobilized pigeons for 30 min. This experiment was conducted using protocol 15-H-034 approved by American Association for Laboratory Animal Science - IACUC.

Nine interspecific crosses were conducted and inspected daily looking for copulation posture and eggs. Five experimental crosses were performed for one female (♀) of *P. chinai* with two males (♂) of *P. howardi*, labeled “♀c × ♂h”; and four crosses were performed for one female of *P. howardi* with two males of *P. chinai*, labeled “♀h × ♂c”. Once the adults F1 emerged, we constituted nine couples in an attempt to produce F2 offspring. We also conducted control crosses for each species, 24 crosses for *P. chinai* and 4 for *P. howardi*. These parental crosses were composed of two females and three males. The eggs of these crosses were used in the morphometric analyses.

### Ecological niche modeling (ENM)

We used data collected in Loja and Manabí provinces (Additional file 1: Table S1). *Panstrongylus chinai* specimens came from 41 communities in Loja Province, and *P. howardi* specimens from 27 communities in Manabí Province. Each locality was georeferenced in decimal coordinates (decimal coordinates have a reasonable error range ± 8–9 m). The ENM was designed to estimate the potential distribution area of each species [75, 76]. Given the suspicion that *P. chinai* and *P. howardi* could be local populations of the same species [13], we used the ENM method to determine if their potential distribution area might show some overlap. Due to the lack of relevant data from Peru, where *P. chinai* occupies a small territory at the border with Ecuador (Loja), we decided to define the entire coastal area up to 2000 masl of Ecuador, excluding Peru, as our calibrated area. We eliminated 4 of the 19 bioclimatic variables to avoid “distortions” to the distribution model [77]. To determine the potential distribution areas for *P. howardi* and *P. chinai*, we used an environmental dataset from the WorldClim data archive [78], selecting a set of 10 “bioclimatic” data layers of 30 arc-seconds (~ 1 km). These data layers were selected after determining that they do not have a climatic correlation with each other. Each data layer’s contribution was high in a jacknife analysis of the model.

Bioclim variables included total monthly precipitation, maximum and minimum annual temperatures and other variables derived from monthly rainfall and temperature. These variables represent annual trends, seasonality and extreme or limiting environmental factors [79].

### Statistical analyses

#### Phenotype: sensilla distribution on antennae

The number and type of antennal sensilla [bristles (BR) thin-walled trichoidea (TH) thick-walled trichoidea (TK) basiconic (BA)] in each segment (Pedi, Flag1, Flag2) were recorded and descriptive statistics were calculated. Homogeneity of variance was checked by the Levene test and normality was checked using the Kolmogorov-Smirnov test. Homoscedastic variables were analyzed using analysis of variance (one-way ANOVA), whereas heteroscedastic variables were analyzed using the Kruskal–Wallis non-parametric test. Univariate analyses of receptor densities were performed using the SPSS package (Statistical Package for Social Sciences) for Windows, version 19.0 (SPSS Inc., Chicago, Illinois). Based on the significant variables, discriminant analyses (one for each sex) between taxa and corresponding cross-validated reclassifications were performed with the CLIC package (version 99; https://xyom-clic.eu).

#### Geometric morphometrics

*Size of wings and heads.* Mean and variance of size were estimated in each sex and compared. Also, using a non-parametric ANOVA based on random sampling with replacement (bootstrap), the amount of sexual size dimorphism (SSD) of *P. chinai* was compared with that of *P. howardi*. In each sex of each species, the linear correlation coefficient (*r*) was computed between head and wing size dimensions of each individual.

*Shape of wings and heads.* Shape variables were the partial warps computed after the Procrustes superimposition of each configuration on the consensus configuration [80]. Shape divergence between taxa was estimated in each sex by the Mahalanobis distance computed from these shape variables. In the same way as for the size variation, the observed Mahalanobis distances were subjected to non-parametric tests.

In addition to the comparison of mean shapes, we compared the variances of shape or metric disparity (DM), as described by Zelditch et al. [81]. We performed shape-based validated reclassifications [82] between species in each sex, using either head or wing shape. As for size, we estimated the shape difference between sexes (“SShD”, for sexual shape dimorphism) and we verified that its importance was similar in both taxa. Using the same non-parametric test as for shape variation between species (see above), we tested in each species the significance of the Mahalanobis distance between sexes. To compare SShD between taxa, a two-factor MANCOVA was used to test shape variation against two main effects (species and sex) and against their interaction. Since shape difference is not only represented by the magnitude of shape change but also by its direction in the morphospace, we adopted the general approach for the statistical comparison of multivariate vectors of phenotypic change as described by Collyer & Adams [83], and applied to the Triatominae by Caro-Riaño et al. [84].

To explore the co-variation of head and wing shape, we computed the Escoufier’s “Rv” coefficient [85]. It is a measure of correlation between two sets of variables, working directly on variance and covariance rather than on correlation matrices [86]. The statistical significance of the Rv coefficient was assessed by randomizing 1000 times the covariance structure between blocks without affecting variance-covariance structure within the blocks, computing each time a pseudo-coefficient and counting the number of times where randomly generated Rv could be equal to or larger than the observed one.

*GM, adults: size and shape*. A multivariate regression was computed with size as independent variable and shape descriptors (partial warps) as dependent variables, and a non-parametric test of statistical significance was applied as described by Good [87]. A multivariate test of significance for “different slopes” *versus* “common slope” model was computed according to the Wilk’s test, for heads and for wings, in both sexes. It is based on the comparison of two matrices derived from residuals of the regression of shape variables against size. The contribution of size to shape divergence between either sexes or species was computed as the coefficient of determination when regressing the (unique) discriminant factor on size.

*GM, eggs: size and shape variation*. Egg size was estimated as the perimeter of the egg contour and compared between groups using non-parametric tests. For shape variable definition, we exclusively used the elliptic Fourier analysis (EFA) [87]. To accurately represent a closed curve, many harmonics may be needed, each with four coefficients, so that the number of variables could be too numerous relative to the number of specimens. The normalized coefficients (NEF) were thus submitted to a principal components analysis (PCA), and the principal components (PCs) were the final shape variables. The Mahalanobis distances between them were used to test for validated reclassification. The contribution of size variation to shape-based discrimination was estimated through the determination coefficient between the first (and unique) discriminant factor and the estimator of size. As for heads and wings, we compared the variance of size and of shape between taxa, we tested the hypothesis of a common allometric model, as well as the contribution of size to shape-based discrimination. All morphometric analyses (size and shape of adults and eggs) were performed using the CLIC package (version 99).

#### Molecular genetics: alignment, model selection and phylogenetic analyses

Alignments were performed using MAFFT [88]. The GTR+I nucleotide substitution model was determined as the best-fitted model by model generator [89], under the Bayesian information criterion (BIC). The maximum likelihood (ML) tree was obtained by PhyML [90], with a nodal support of 1000 bootstrap pseudo-replicates. The phylogenetic trees were visualized and edited using FigTree [91], and ggtree [92]. Intra- and interspecific sequence divergence for the *cytb* gene was assessed with Kimura 2-parameter (K2P) distance model in MEGA v6 [93].

#### Experimental crosses

Mean and standard deviation of pre-oviposition time were computed for each taxon and for hybrids. We also estimated the percentage of successful crosses, average number of eggs obtained by female, developing time and percentage of viable eggs. The latter was calculated as the ratio of total hatched eggs over total laid eggs. These analyses were performed using the SPSS package, version 19.0.

#### Ecological niche modeling

We used MaxEnt 3.4.1 [94] to determine the potential distribution of each species; the software is useful for estimating a distribution across geographical space [95, 96], based on the presence or absence of some species. It used bioclimatic layers belonging to the Bioclim world climate database. The results of ecological niche modeling (ENM) analyses were exported to the ArcGIS (Geographic Information System) Software by Esri (Environmental Systems Research Institute) for Windows, version 10.3. (Release 10. Redlands, California). Because the number of points from presence was low, we utilized 10,000 points as background. To measure the robustness of model estimation, we used a cross-validation procedure, and a random seed with 10 replicates. To evaluate the validity of the model we used the “area under curve” (AUC estimation. Finally, we also estimated the index of niche overlap “D” using the software ENM tools for Windows, version 1.4.4 [94].

## Results

### Antennal phenotype

Table 2 shows the means and standard deviations for the number of antennal sensilla in both sexes and in each species without discriminating by habitat. Significant differences were detected between taxa for TH from all examined segments of the antenna (Pedi, Flag1 and Flag2). For males only, the BR of the pedicel also showed significant differences. For the two species, no sexual dimorphism in the antennal phenotype could be observed. Multivariate analyses (one for each sex) using as input the significant variables (4 for males, 3 for females) did not reveal significant Mahalanobis distances *(P* > 0.05) between the two species. Cross-check classification resulted in relatively low scores (from 56% to 68%).

**Table 2:** Means (± standard deviations) of the number of bristles (BR), thin-walled trichoid (TH), thick-walled trichoid (TK), and basiconica (BA) of the antenna of *Panstrongylus chinai* and *P. howardi*

Species	Sex	BR	TK	TH	BA
		Pedi*M	Pedi	Pedi*MF	Pedi
*P. chinai*	Males (*n* = 29)	84.5 ± 11.4	3.4 ± 2.8	278.8 ± 74.4	5.51 ± 3.21
	Females (*n* = 16)	102.6 ± 8.2	6.1 ± 4.8	101.7 ± 25.5	4.6 ± 2.7
*P. howardi*	Males (*n =* 15)	101.1 ± 17	3.2 ± 4.9	257.5 ± 83.9	6.5 ± 2.7
	Females (*n =* 8)	101.5 ± 11.3	2.4 ± 3.2	214 ± 37.2	5.3 ± 1.3
		Flag1	Flag1	Flag1*MF	Flag1
*P. chinai*	Males (*n =* 29)	29.1 ± 6.4	77.4 ± 28.2	157.4 ± 31.9	40.5 ± 8.3
	Females (*n =* 16)	32.7 ± 6.3	51.6 ± 17.1	157.6 ± 48.8	29.2 ± 8.9
*P. howardi*	Males (*n =* 15)	31.1 ± 5.9	79.5 ± 27.1	181.6 ± 41.1	43.2 ± 13.5
	Females (*n =* 8)	35.1 ± 2.3	57 ± 18.5	166.8 ± 38.2	28.4 ± 4.9
		Flag2	Flag2	Flag2*MF	Flag2
*P. chinai*	Males (*n =* 29)	19.2 ± 4.1	38.1 ± 15.3	133.2 ± 36.5	31.7 ± 13.6
	Females (*n =* 16)	22.3 ± 3.9	24.1 ± 8.2	157.8 ± 38.8	23.3 ± 9.2
*P. howardi*	Males (*n =* 15)	19.2 ± 3.9	42 ± 15.4	158.5 ± 42.2	30.1 ± 8.6
	Females (*n =* 8)	24.5 ± 3.4	37.4 ± 12.2	183 ± 22.9	27.3 ± 7.9

*Abbreviations*: *MF, statistically significant between taxa in both sexes (males and females) for TH in Pedicell; Pedi, Pedicell; Flag1, flagellum 1; Flag2, flagellum 2; *M, in males only for BR in Pedicell; *n*, number of specimens in each group

### Geometric morphometrics: adults

**Fig. 5:**
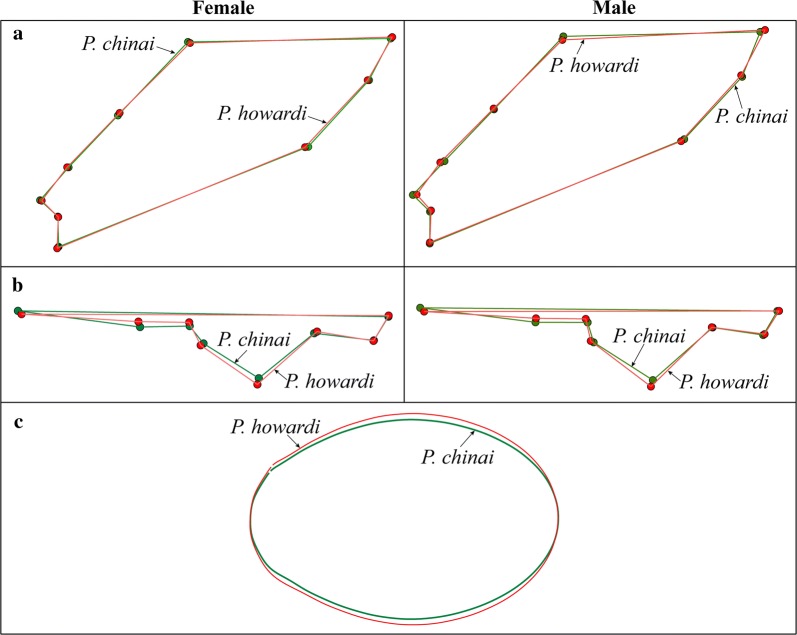
Shape of wings, head and eggs. **a** Wing shape differences between taxa, according to sex. **b** Difference in head shape between taxa, according to sex. **c** Egg shape differences between outlines for *P. chinai* and *P. howardi*

For head and wing shape (Fig. 5a, b), a significant difference was generally found between taxa for males and females (Table 3). The reclassification produced better results when based on wing shape relative to head shape, with scores generally better for males (85%, 92%, 76%, 80%) than for females (93%, 66%, 73%, 77%). The variance of shape (metric disparity, MD) did not show statistically significant differences between taxa, except for the variance of head shape, larger in male *P. howardi* (*P* = 0.012). Within each taxon, the variance of head shape (MD range of 10.1–16.8) was approximately two times the variance of wing shape (MD range of 6.8–8.6).

**Table 3:** Statistics performed on the same sample sizes for heads and wings: females (18 *Panstrongylus chinai vs* 15 *P.howardi*); males (25 *P. chinai vs* 21 *P. howardi*)

Metric divergence between taxa	Heads		Wings	
	Females	Males	Females	Males
Comparisons of size				
Mean	*P* < 0.0001*	*P* < 0.0001*	ns	0.006*
Variance	ns	ns	ns	ns
Comparisons of shape				
Mahalanobis	*P* < 0.0001*	*P* < 0.0001*	*P* = 0.002*	*P* < 0.0001*
Percent contribution of size	52	58	2	18
Validated reclassification *P. chinai*	77%	80%	66%	92%
Validated reclassification *P. howardi*	73%	76%	93%	85%
Metric disparity *P. chinai*	16.8	12.2	8.6	7.9
Metric disparity *P. howardi*	10.1	16.4	7.7	6.8
Metric disparity, comparisons	ns	(*)	ns	ns

*P < 0.05

*Notes*: “Contribution of size to shape divergence” is the coefficient of determination after regressing the discriminant factor on size variation; “Validated reclassification”, scores of correctly assigned specimens based on shape variation

*Abbreviations*: P, risk of error estimated according to non-parametric technique based on permutation tests (1000 runs); ns, not significant; Mahalanobis, shape differences between taxa according to the Mahalanobis distances

**Fig. 6:**
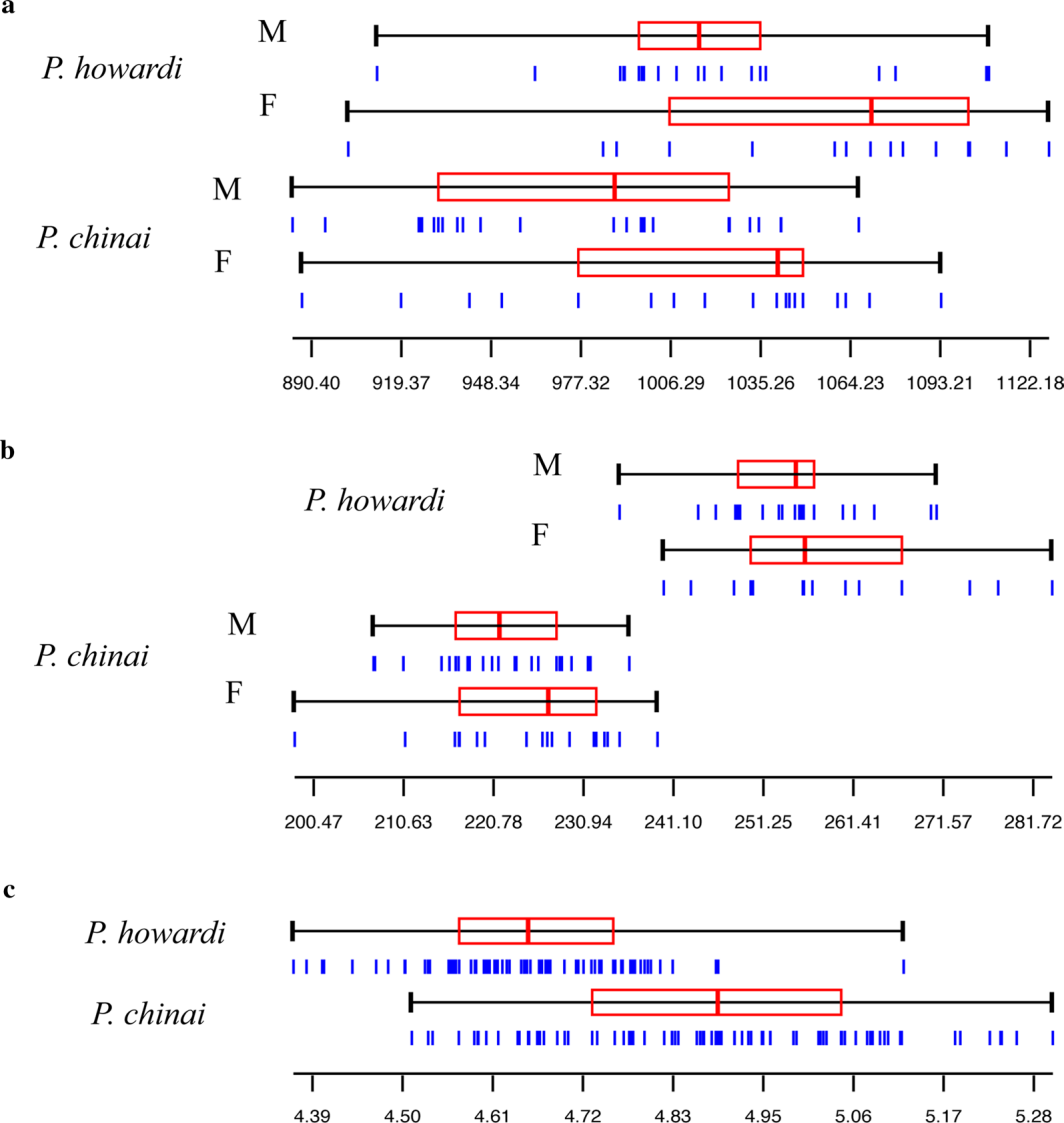
Size variation of wings, heads and eggs. **a** Centroid size of wings (pixels) for taxa and sexes. **b** Centroid size of heads (pixels) for taxa and sexes. **c** Perimeter of the external contour of eggs (mm) for *P. chinai* and *P. howardi*

Size variation was illustrated for wings, heads and eggs by quantile boxes (Fig. 6). In both taxa, sexual dimorphism of size was not detected for heads, and was nearly significant for wings (Table 4). For the head, shape variation between sexes was found to be significant in both species. For the wing, shape variation was significant between sexes in *P. chinai* only. However, there was no significant difference in the amount of sexual dimorphism between taxa for head or wings (Table 4). Actually, the Procrustes distances between sexes were very similar between taxa for heads (0.036 for *P. chinai* and 0.031 for *P. howard*) and for wings (0.017 and 0.011, respectively).

**Table 4:** Sexual dimorphism within and between species

Sexual dimorphism	Species	Heads *P*-value (F *vs* M)	Wings *P*-value (F *vs* M)
Sexual size dimorphism (SSD)			
Mean	*P. chinai*	ns	0.030*
	*P. howardi*	ns	ns
Variance	*P. chinai*	ns	ns
	*P. howardi*	0.040*	ns
Comparisons of SSD between taxa (bootstraps)			
Mean		ns	ns
Variance		ns	ns
Sexual shape dimorphism (SShD)			
Mahalanobis	*P. chinai*	< 0.0001*	0.001*
	*P. howardi*	< 0.0001*	ns
Comparison of SShD between taxa			
dc	*P. chinai*	0.036	0.017
dh	*P. howardi*	0.031	0.011
dc-dh		ns	ns
Angle dc/dh		ns	ns

*P < 0.05

*Abbreviations*: P, risk of error estimated according to non-parametric technique based on permutation tests (1000 runs); ns, not significant; Mahalanobis, shape differences between sexes according to the Mahalanobis distances; dc, the magnitude of shape change between sexes according to the Procrustes distance for *P. chinai*; dh the magnitude of shape change between sexes according to the Procrustes distance for *P. howardi* (“dh”); Angle dc/dh, change in the direction of shape differences between sexes; F, female; M, male

Within each species and in each sex, for heads and for wings, the non-parametric multivariate regression of shape against size variation was not significant. As expected, for heads or for wings, both taxa shared a common allometric axis, suggesting similar patterns of growth (details not shown). A much stronger contribution of size to head shape divergence (52–58%) was found, relative to the contribution of size to wing shape difference (2–18%). Head shape divergence was dominated by size variation, and the reclassification of specimens based on head shape variation was less satisfactory than that based on wings (Table 3).

The covariation of head and wing shape was very similar between taxa in both sexes (0.19 for female *P. chinai* and 0.22 for female *P. howardi*, 0.29 and 0.35 for corresponding males). Contrasting with this similarity, head size was differently correlated with the wing size according to the species (Table 5). The correlation of size variation between head and wing was high (0.81 in males and 0.84 in females) and significant (*P* < 0.001) in *P. chinai* but much lower in *P. howardi* (0.25 in males and 0.52 in females), where it was only significant for females.

**Table 5:** Correlation coefficients between head and wing variation

Species	Size / Shape	Females	*P*-value	Males	*P*-value
*P*. *howardi*	Size (r)	0.52	0.024*	0.25	0.140
	Shape (Rv)	0.23	0.528	0.35	0.040
*P. chinai*	Size (r)	0.84	< 0.001*	0.81	< 0.001*
	Shape (Rv)	0.20	0.744	0.29	0.063

*Notes*: For size correlation coefficients (r), *P*-value was computed according to the Studentʼs t-test. For shape correlation coefficients (Rv), which are here the Escoufier’s coefficient (see Methods), *P*-values are the proportion of pseudo-coefficients equal or larger than the observed coefficient after non-parametric tests (1000 runs)

### Geometric morphometrics: eggs

While *P. howardi* showed a larger size than *P. chinai* for both heads and wings, eggs in this species were significantly smaller. Egg shape variation between taxa was very significant (Table 6), with outline divergence visible without image amplification (Fig. 5c). The variance of size and shape showed significantly higher values for *P. chinai*, and, contrary to the adult morphometric variation, these values were different between taxa. Also contrary to the adults, the common allometric model was rejected (*P* = 0.040, details not shown). Finally, the reclassification scores based on the contour shape among taxa were excellent: 97% (76/78) for *P. chinai* and 94% (71/75) for *P. howardi* (Table 6). The Mahalanobis distance (6.27) was highly significant (*P* < 0.0001), and the influence of size on this shape-based distinction was 19%.

**Table 6:** Statistical comparisons (*P*-value, error risk) of egg metric properties between *Panstrongylus chinai* and *P. howardi*

Size / Shape	Statistical measures	*P. chinai*	*P. howardi*	*P*-value
Size (perimeter)	Mean	4.88 ± 0.19	4.66 ± 0.12	< 0.0001
	Variance	0.035	0.015	< 0.0001
Shape	Validated reclassification	97% (76/78)	94% (71/75)	
	Metric disparity	0.00059	0.00039	< 0.0100

### Cytogenetics

**Fig. 7:**
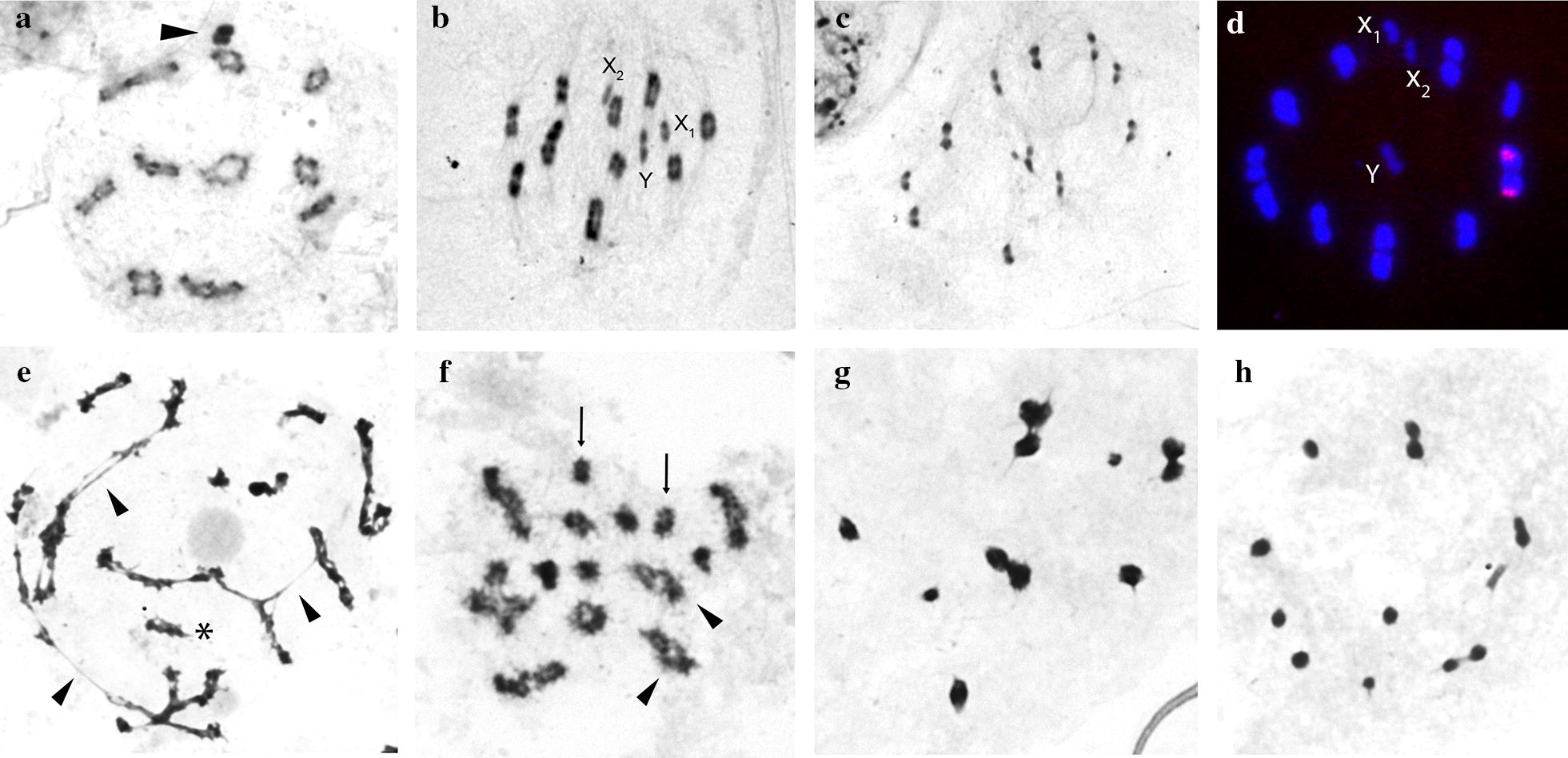
Male meiosis of *Panstrongylus howardi* (2n = 20 A + X_1_X_2_Y) (**a**–**d**) and male interspecific hybrids resulting for the crosses between *P. howardi* (♀) and *P. chinai* (♂) and the reverse cross (**e**–**h**). **a**–**c** C-banding technique. **d** Fluorescent *in situ* hybridization (FISH) with *45S* ribosomal DNA probe. **e**–**h** Giemsa stain. **a** Diplotene. The ten autosomal bivalents are clearly observed and showing heterochromatic C-blocks in both chromosomal ends. The three sex chromosomes remain associated (arrowhead). **b** Metaphase I. The ten autosomal bivalents and the three sex chromosomes (univalents) appear clearly separated. **c** Metaphase II. Typical chromosome configuration seen in Triatominae species: the three sex chromosomes in the center of a ring formed by the autosomes. The X1 and X2 chromatids segregate to the same pole, while the Y chromatid migrates to the opposite one. **d** Metaphase I. The *45S* rDNA signals are located in one of the largest autosomal bivalent. **e** Paquitene stage. Chains of bivalents, univalents and even chromosomal fragments are observed, products of the alteration of chromosomal pairing. **f** Late diplotene. Associated bivalents (arrowheads) and univalents (arrows) can be observed. Metaphases I (**g**) and Metaphases II (**h**) altered, with deficiency or excess of autosomes and sex chromosomes

The comparative analyses of *P. chinai* and *P. howardi* from Ecuador studied here with previously published data for *P. chinai* from Peru [97], showed that the two species have the same chromosomal characteristics. C-banding revealed they have the same chromosome diploid number constituted by 23 chromosomes (2n = 20 autosomes + X_1_X_2_Y) in males and similar amount of heterochromatic regions. The ten autosomal bivalents exhibited a C-positive heterochromatic block in both chromosomal ends (Fig. 7a, b). The Y sex chromosome was totally heterochromatic while the X_1_ and X_2_ sex chromosomes were the smallest of the complement and presented an intermediate staining (Fig. 7b, c). According to the FISH technique, the *45S* ribosomal DNA cluster was located in the largest autosomal pair in both taxa (Fig. 7d).

The meiotic analysis of two male adults (F1) obtained from the two interspecific crosses showed important anomalies in the chromosomal behavior. In pachytene, association of different bivalents forming chains, univalents (consequence of the lack of pairing between homologous chromosomes) and chromosome fragments are observed, all of them representing products of alterations in chromosomal pairing (Fig. 7e). During late diplotene, associated bivalents and univalents were observed (Fig. 7f). The proportion of bivalents and univalents varied not only between the two analyzed individuals, but also between cells of the same specimen. These abnormalities produced metaphases I (Fig. 7g) or metaphases II (Fig. 7h) with variable numbers of autosomes and sex chromosomes. All metaphases I (Fig. 7g) and metaphases II (Fig. 7h) were aneuploid, with deficiency or excess of chromosomes. Normal first and second metaphases were not observed.

### Molecular genetics

**Fig. 8:**
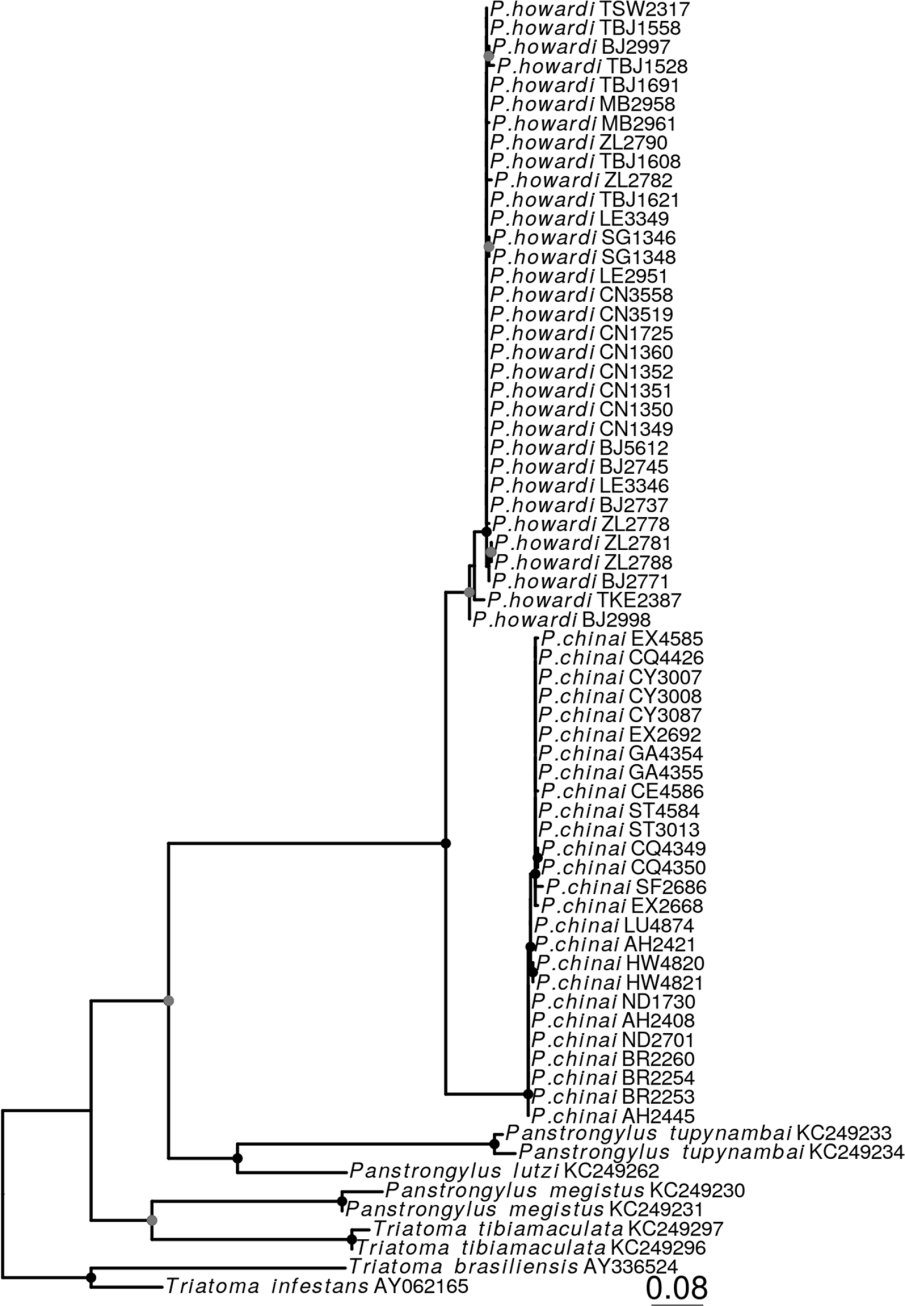
Maximum likelihood tree based on analysis of *cytb* gene sequences of 59 individuals of *Panstrongyluschinai* and *P. howardi* available on GenBank. Six species (3 *Panstrongylus* spp. and 3 *Triatoma* spp.) were included for comparative purposes. Nodes indicated with a gray circle are those whose support is higher than 50 bootstrap pseudoreplications and with black circle higher than 70. ID code of specimens is indicated for each terminal

Intra-species genetic K2P distances for *P. chinai* (26 individuals) and *P. howardi* (33 individuals) varied between 0–0.94% (average 0.32%) and 0–1.9% (average 0.28%), respectively (Additional file 2: Table S2). Genetic distance between *P. chinai* and *P. howardi* was 7.82% on average (range 6.25–8.73%). Lastly, *P. chinai* and *P. howardi* distance with the other three *Panstrongylus* species ranged between 17.93–24.11%. In the ML tree (Fig. 8), all nodes showed high support (> 70) and strongly supported the monophyly of *P. chinai* and *P. howardi* (bootstrap value 100).

### Experimental crosses

**Fig. 9:**
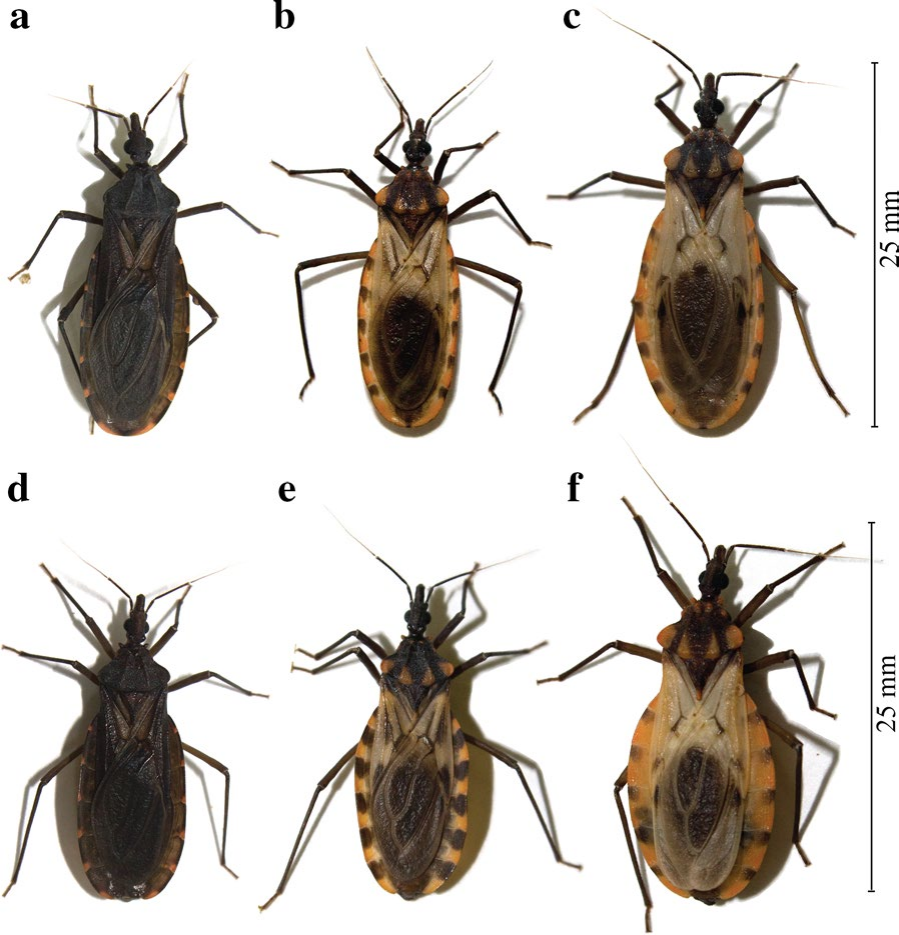
Adults of *Panstrongylus chinai*, *P. howardi* and the hybrids from crosses between them. **a** Male *P. chinai*, a species distributed in Loja Province (Ecuador) and in the north of Perú. **b** Male hybrid of the crosses between females of *P. chinai* and males of *P. howardi*. **c** Male *P. howardi*, an endemic species from Manabí Province (Ecuador). **d** Female *P. chinai*. **e** Female hybrid of the cross between female *P. chinai* and male *P. howardi*. **f** Female *P. howardi*

Copulation and viable eggs were observed in seven out of the nine interspecific couples (F1 generation). Most of the couples were successful, 4 out of 5 of the ♀c × ♂h crosses and 3 out of 4 of the ♀h × ♂c crosses. All adult F1 hybrids of each sex showed a general color typical of the *P. howardi* phenotype, while they showed a general size and shape closer to the *P. chinai* phenotype (Fig. 9). In spite of the daily examination of the crosses between F1 hybrids, no copulation was observed. Eggs appeared in 6 out of the 9 couples, all of them empty, with no visible embryo; these eggs promptly shrank and never hatched.

The pre-oviposition period was slightly lower when the females were crossed with males of the same species (parental crosses) (Table 7). The average number of eggs was the greatest for *P. howardi*, but their viability was two times lower than for other crosses (Table 7). No significant differences were observed in the time of development between the parental crosses with the interspecific crosses.

**Table 7:** Successful (offspring-producing) crosses, mean (± SD) number of eggs per female, percentage of viable eggs, and mean (± SD) development time of the eggs obtained per cross among *Panstrongylus howardi* and *P. chinai* (parental crosses) and interspecific crosses

Species/experimental crosses	No. of crosses	Pre-oviposition time (days)	Mean no. of eggs	Viable eggs (%)	Development time (days)
*P. chinai*	24	11 ± 5.65	34	88.88	29.66 ± 1.47
*P. howardi*	4	15 ± 1.41	55	44.03	29.28 ± 1.59
♀c × ♂h	4	14 ± 2.00	52	80.4	27.28 ± 0.99
♀h × ♂c	5	18 ± 6.56	40	89.08	29.24 ± 1.26

*Abbreviations*: ♀c × ♂h, female *P. chinai* × male *P. howardi*; ♀h × ♂c, female *P. howardi* × male *P. chinai*

### Ecological niche modeling

**Fig. 10:**
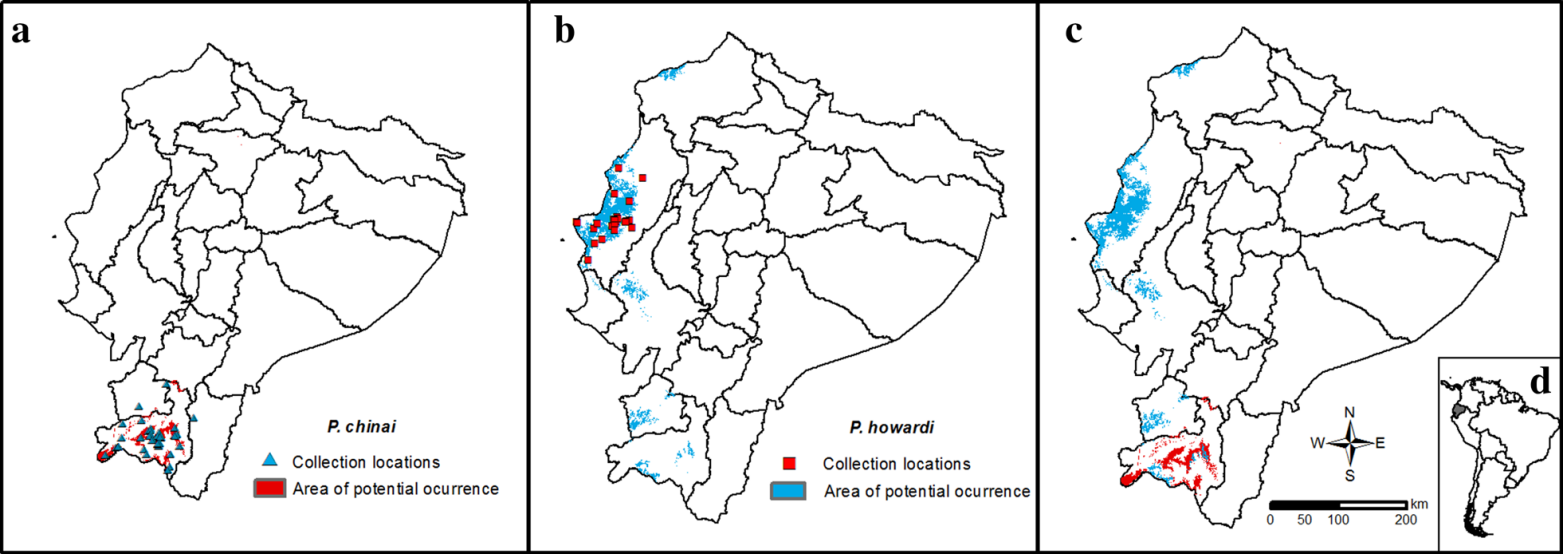
Ecological niche modeling. **a** Collection locations of *Panstrongylus chinai* (triangles), and predicted distribution of this species, which includes Loja, Azuay and El Oro provinces. **b** Collection locations of *P. howardi* (squares) in Manabí province, and the predicted distribution of this species, which includes Esmeraldas, Guayas, El Oro, and some parts of Loja provinces. **c** Combined predictive models of *P. chinai* (orange) and *P. howardi* (blue), showing the overlapping potential areas in Loja province. *d* Map of South America showing the location of Ecuador

The ENM model for *P. chinai* adequately predicted the current distribution of this species but also predicted its occurrence in the north of the province of Loja, between Loja, Azuay and El Oro provinces (Fig. 10a). Figure 10b shows the distribution of *P. howardi* in Manabí Province, with a geographical expansion of this species to Esmeraldas, Guayas, El Oro and some parts of Loja. Figure 10c provides the predictive models of *P. chinai* and *P. howardi* together, showing the overlapping potential areas of *P. howardi* with *P. chinai* in Loja Province. Thus, Loja and Manabí apparently share environmental characteristics suitable for the colonization by both species. For *P. chinai*, the model fit had an AUC of 98%, while for *P. howardi* the AUC was 96%. The index of the niche overlap was *D* = 0.140 with *P* > 0.05, suggesting similarity rather than equivalence.

The distribution model for *P. chinai* showed that the mean diurnal range (maximum temperature − minimum temperature, BIO2; Table 8) significantly contributed (77.7%) to the presence of this species, together with the annual mean temperature (BIO1, 7.6%) and the temperature seasonality (BIO4, 5.8%; Table 8). The model fit had an AUC of 98%. The distribution model for *P. howardi* showed that BIO12 (annual precipitation) (Table 8) had the highest contribution (61.3%), followed by the temperature seasonality (BIO4, 17.3%) and the annual mean temperature (BIO1, 14.2%; Table 8).

**Table 8:** Bioclimatic variables used for the ecological niche modeling (ENM)

Bioclimatic variable	Variable contribution (%)	
	*P. howardi*	*P. chinai*
BIO2: Mean diurnal range (mean of monthly (max temp − min temp))	1.1	77.7
BIO1: Annual mean temperature	14.2	7.6
BIO4: Temperature seasonality (standard deviation × 100)	17.3	5.8
NDVI	5.9	4.3
BIO14: Precipitation of driest month	0.1	1.7
BIO7: Temperature annual range (BIO5-BIO6)	0	1.5
BIO13: Precipitation of wettest month	0	1.3
BIO3: Isothermality (BIO2/BIO7 × 100)	0	0
BIO12: Annual precipitation	61.3	0
BIO15: Precipitation seasonality (coefficient of variation)	0.1	0

## Discussion

Species of the Triatominae display a high degree of morphological plasticity [64]. Due to this, entomologists face situations where morphological differences exist between groups (mainly size or/and color variation) without clear genetic support, and others where genetic variation seems important without morphological discriminating traits, or even without established reproductive isolation [98]. Thus, morphological or genetic variation should not lead *per se* to the erection of a new species. In the spirit of integrative taxonomy, we followed the recommendations of Bargues et al. [19, 21]: “a multidisciplinary approach would converge to the hypothesis of an evolutionary lineage, or, conversely, to the idea of an intraspecific variation”. In the speciation process, the different biological properties examined here are not expected to evolve at the same time and in a regular order [20, 99], so that opposite trends could be expected. We observed indeed incongruous results preventing us to modify the current taxonomic status of *P. chinai* and *P. howardi*.

Most of the specimens (94%) were collected in domestic and peridomestic environments; however, most *P. chinai* (95%) were found inside houses while most *P. howardi* (90%) were collected in peridomestic habitats. In addition to a behavioral difference between the two species, this observation probably reflects the difference in habitats between Loja and Manabí. Houses in Loja are made of adobe, where the bugs can hide [62], while houses in Manabí have cane walls not suitable for bug infestation [11, 63]. Different rates of trypanosomatid infection were reported for these two taxa, with *T. cruzi* prevalence of 57.5% for *P. howardi* [7] and 12.8% for *P. chinai* [62].

According to a cladistic analysis based on morphological characters, Lent & Wygodzinsky [3] suggested that *P. chinai* and *P. howardi* constitute a separate clade of two closely related species in the genus *Panstrongylus*. What justified their distinct species status was mainly the clear-cut difference in color. Many species in the Triatominae may be recognized thanks to their color patterns. In the case of *P. chinai* and *P. howardi*, the color difference could suggest the existence of dark or melanic forms as already observed within some other species, such as *T. infestans* [100], *R. stali* [26] and *R. nasutus* [101]. Such melanization is not necessarily suggestive of distinct species [29, 102].

The antennal phenotype has proven to be a useful tool for the taxonomic comparison among species of the Triatominae [103]. Between geographically separate populations, significant differences may also arise, as it was the case for wild and domestic populations of *T. infestans* [102], *R. ecuadoriensis* [5] and six other *Triatoma* species [104]. The differences disclosed here between the antennae of *P. howardi* and *P. chinai* were lower than those found between populations of *R. ecuadoriensis* [5].

We detected a lower morphometric variation of the head and wings than expected for distinct species. Altogether, the amount of metric divergence could be in conformity with local populations of the same species. Size difference is a common finding between local populations. Despite living in a warmer climate, the Manabí specimens tended to be larger, which aligns with Bergmann’s rule. This rule also applies to intraspecific populations as a species criterion by Marcondes et al. [105]. However, this argument may not be as relevant amongst populations located close to the equatorial line [106]. For instance, the Bergmann’s rule was contradicted for populations of *R. ecuadoriensis* collected in the same provinces, Loja and Manabí [5]. The clear-cut difference in the correlations of head and wing size is not an argument suggesting different species; it has been observed between populations of the same species from different habitats [107]. Shape differences are expected between species, but they are also common between populations, either due to isolation or adaptation to different environments, or both. Shape changes *per se* cannot distinguish the variation due to simple genetic drift between isolated subpopulations from that due to evolutionary divergence between species. However, the relatively low reclassification scores, the lack of difference in the magnitude of sexual dimorphism, the apparent lack of difference in the covariation of head-wing shape, and a common allometric axis do not advocate for very different organisms.

Metric differences of the eggs between taxa, especially shape differences, were clearly more pronounced than those of head or wing. By comparing laboratory lines reared under the same conditions, fed on the same blood source at the same frequency, using eggs at similar developmental stages and positions, we could reduce the possible environmental influence on shape variation. However, the metric differences between eggs of both taxa could be attributed in part to the relatively restricted spatial and temporal sampling of one of them. The eggs of *P. howardi* were collected from a single community, on the same day. As a consequence, the *P. howardi* sample could provide a truncated representation of the species variability, as suggested by its lower variance of size and shape (Table 6). However, another reason for the clear-cut difference found between *P. howardi* and *P. chinai* could be a true evolutionary difference.

C-heterochromatin distribution and the chromosome position of ribosomal clusters are the principal cytogenetic traits to aboard the analysis of karyotype differentiation in the Triatominae [42, 43]. In several triatomine groups, such as *infestans* and *sordida* subcomplexes, closely related species exhibit striking differentiation for these cytogenetic markers. However, in other subcomplexes such as *phyllosoma* and *brasiliensis*, valid species did not present cytogenetic differentiation [42]. Therefore, the chromosomal identity found here between *P. chinai* and *P. howardi* does not mean that they should be the same species (Fig. 7a–d). In fact, the chromosomal analysis of the hybrids showed significant alterations in the chromosome pairing of the bivalents which reveals deep chromosomal differences in the parental species not detected with the techniques applied here (Fig. 7e–h). In this way, the meiotic process of the hybrids does not end correctly so that viable gametes are not formed and, as a consequence, these hybrid individuals are infertile. Thus, the meiotic analysis of F1 hybrids strongly suggests that *P. chinai* and *P. howardi* are distinct species.

The *cytb* phylogenetic analyses strongly suggest that *P. chinai* and *P. howardi* are included in the same clade, representing sister species (Fig. 8). Intraspecific average distances were 0.32% and 0.28% for *P. chinai* and *P. howardi*, respectively. These distances are within the expected range for differentiation within a species, which indicates that the species identification for each individual was correct with this marker. The average distance value K2P (7.8%) between *P. chinai* and *P. howardi* is slightly higher than that reported to separate other closely related triatomine species (e.g. 7.5% for the *cytb* marker) [46]. Thus, our molecular data support considering *P. chinai* and *P. howardi* as two separate sister species, in agreement with the suggestion by Barnabe et al. [108].

The hypothesis of a single species was not supported by our experimental crosses and the chromosomal analyses of the F1 hybrids. The main results of our crossing experiments were: (i) the ease to obtain a viable F1 offspring between the two species studied; and (ii) the failure to obtain an F2 offspring due to the lack of copulation between the F1 hybrids. Inter-specific hybrid generation (adult F1), both naturally and experimentally, is a common phenomenon in the Triatominae, and occurs between both closely and evolutionarily distant species of the genera *Meccus*, *Rhodnius* and *Triatoma* [25]. The ease of obtaining inter-specific F1 adults reveals that several pre-zygotic isolation mechanisms can be easily avoided by experimental hybridization. For example, the morphology of the genital structures does not represent an important barrier to mating that prevents interspecific crosses in some Triatominae. However, the great majority of these hybrids are infertile, unable to produce viable offspring [49]. In the Triatominae, interspecific hybrids can be totally fertile or have different degrees of infertility [25]. In the species studied here, the lack of copulation among the F1 hybrids and their inability to produce viable gametes (chromosome studies in males) reveal that different pre-zygotic isolation mechanisms are acting which lead to a total infertility of the F1 hybrids. In conclusion, our experimental crosses and the chromosome studies of the F1 hybrids strongly suggest that both *Panstrongylus* taxa are separate species.

The ENM correctly predicted the current distribution of *P. chinai* and *P. howardi*, but also predicted other locations with a partial overlap. This overlap, restricted to a few sites, suggests that both species share similar not equivalent niches. The ENM analyses suggest that both taxa could be different species.

## Conclusions

Overall, antennal morphology, geometric morphometrics of the head and wing shape, and cytogenetic analysis did not indicate distinct differences between *P. howardi* and *P. chinai*. Conversely, geometric morphometrics of the eggs, ENM analyses, molecular phylogeny, and crossing experiments (including chromosome analyses of the hybrids), in addition to the color pattern and current distribution, support the hypothesis that these bugs are separate species. We conclude that *P. howardi* and *P. chinai* should not be synonymized, they represent two valid closely related species.

## Supplementary information

**Additional file 1: Table S1.** Geographical location and altitude of studied communities in Loja and Manabí provinces, Ecuador.

**Additional file 2: Table S2.** Genetic 2k-p distances among *Panstrongylus chinai* (26 individuals), *P. howardi* (33 individuals), *P. megistus* (2 individuals), *P. tupynambai* (2 individuals), *P. lutzi* (1 individual), *Triatoma tibiamaculata* (2 individuals), *T. infestans* (1 individual) and *T. brasiliensis* (1 individual).

## Data Availability

All data generated or analyzed during this study are included within the article and its additional files. The newly generated sequences were submitted to the GenBank database under the accession numbers JX400933-JX401001.

## Abbreviations

APH: antennal phenotype
AUC: area under curve
BA: basiconic
BR: bristles
CYT: cytogenetics
D: index of niche overlap
EC: experimental crosses
EFA: elliptic Fourier analysis
ENM: ecological niche modelling
FISH: fluorescence *in situ* hybridization
Flag1: flagellum 1
Flag2: flagellum 2
GM: geometric morphometrics
masl: meters above sea level
MD: metric disparity
MG: molecular genetics
NEF: normalized elliptic Fourier coefficients
NV: fifth-instar nymphs
Pedi: pedicel
PLS: partial least square
Rv: Escoufierʼs coefficient
SSD: sexual size dimorphism
SShD: sexual shape dimorphism
TH: thin-walled trichoidea
TK: thick-walled trichoidea
